# Annual Incidence of Snake Bite in Rural Bangladesh

**DOI:** 10.1371/journal.pntd.0000860

**Published:** 2010-10-26

**Authors:** Ridwanur Rahman, M. Abul Faiz, Shahjada Selim, Bayzidur Rahman, Ariful Basher, Alison Jones, Catherine d'Este, Moazzem Hossain, Ziaul Islam, Habib Ahmed, Abul Hasnat Milton

**Affiliations:** 1 Department of Medicine, Shaheed Suhrawardy Medical College, Dhaka, Bangladesh; 2 Department of Medicine, Sir Salimullah Medical College, Dhaka, Bangladesh; 3 School of Public Health and Community Medicine, Faculty of Medicine, University of New South Wales, Sydney, New South Wales, Australia; 4 School of Medicine, University of Western Sydney, Sydney, New South Wales, Australia; 5 School of Medicine and Public Health, Faculty of Health, Centre for Clinical Epidemiology and Biostatistics (CCEB), University of Newcastle, Newcastle, Australia; 6 Directorate General of Health Services (DGHS), Mohakhali, Dhaka, Bangladesh; 7 Department of Community Medicine, National Institute of Preventive and Social Medicine, Dhaka, Bangladesh; 8 Mymensingh Medical College, Mymensingh, Bangladesh; Liverpool School of Tropical Medicine, United Kingdom

## Abstract

**Background:**

Snake bite is a neglected public health problem in the world and one of the major causes of mortality and morbidity in many areas, particularly in the rural tropics. It also poses substantial economic burdens on the snake bite victims due to treatment related expenditure and loss of productivity. An accurate estimate of the risk of snake bite is largely unknown for most countries in the developing world, especially South-East Asia.

**Methodology/Principal Findings:**

We undertook a national epidemiological survey to determine the annual incidence density of snake bite among the rural Bangladeshi population. Information on frequency of snake bite and individuals' length of stay in selected households over the preceding twelve months was rigorously collected from the respondents through an interviewer administered questionnaire. Point estimates and confidence intervals of the incidence density of snake bite, weighted and adjusted for the multi-stage cluster sampling design, were obtained. Out of 18,857 study participants, over one year a total of 98 snake bites, including one death were reported in rural Bangladesh. The estimated incidence density of snake bite is 623.4 / 100,000 person years (95% C I 513.4–789.2 /100,000 person years). Biting occurs mostly when individuals are at work. The majority of the victims (71%) receive snake bites to their lower extremities. Eighty-six percent of the victims received some form of management within two hours of snake bite, although only three percent of the victims went directly to either a medical doctor or a hospital.

**Conclusions/Significance:**

Incidence density of snake bite in rural Bangladesh is substantially higher than previously estimated. This is likely due to better ascertainment of the incidence through a population based survey. Poor access to health services increases snake bite related morbidity and mortality; therefore, effective public health actions are warranted.

## Author Summary

Snake bite is one of the major causes of morbidity and mortality in many rural tropical areas. As a neglected public health problem, estimate of the risk is largely unknown. However, the associated personal and economic impact of snake bite is substantial across developing countries. This national survey investigated the risk and consequences of snake bite among the rural Bangladeshi population. We surveyed 18857 individuals from 24 out of 64 districts in Bangladesh where 98 snake bites including one death were reported. The estimated incidence density of snake bite is 623.4/ 100,000 person years (95% CI: 513.4–789.2/100,000 person years). Biting occurs mostly when individuals are at work. The majority of the victims (71%) received snake bites to their lower extremities. Eighty-six percent of the victims received some form of management within two hours of snake bite, although only three percent of them went directly to either a medical doctor or a hospital. The observed rate of snake bite in rural Bangladesh is substantially higher than anticipated. This coupled with poor access to health services led to an increase in related morbidity and mortality. An improvement in public health actions is therefore warranted.

## Introduction

Snake bite particularly in the rural tropics is a major cause of mortality and morbidity, and it has a significant impact on human health and economy through treatment related expenditure and loss of productivity [1]. Snake bite is the single most important cause of envenoming worldwide and results in substantial mortality in many parts of Africa, Asia, and the Americas [2]. Snake bite is significantly neglected as a public health problem in the world as evidenced by the lack of available incidence data from most of the rural tropics where snake bites occur frequently. Global snakebites (envenomings) incidence has been estimated as 500,000 and mortality between 30000–40000 per year [3]. Chippaiux estimated that venomous snakes cause 5.4 million bites, approximately 2.5 million envenomings and over 125,000 deaths worldwide annually [4]. White estimated more than three million bites per year resulting in more than 150,000 deaths [5]. Details of the methods used to estimate these numbers have not been clearly described. More recently Anuradhani et al reported that, globally at least 421,000 envenomings occur annually, but this may be as high as 1,841,000 [6]. According to this estimate, the highest numbers of envenomings are estimated for South Asia (121,000) followed by South East Asia (111,000), and East Sub-Saharan Africa (43000). Global estimates of snakebite envenomings and deaths seem to be more accurate than previous estimates due to improved study methodology. However, this data may be inaccurate because of assumptions used in the calculations, lack of information relating to snake bites and related deaths in rural tropics. It is likely that the true numbers of these events may be substantially different from the estimates presented in this report.

The true incidence of snake bite in rural Bangladesh is largely unknown. Previously, an incidence of 4.3 snake bites per 100,000 populations was reported with approximately 2000 deaths occurring annually in Bangladesh [7]. This estimate is based on data from a small study. During 1988–89, a small survey was conducted in 50 Upazillas (sub-districts) of Bangladesh that recorded 764 episodes of snakebite, of which 168 (22%) died [8]. Due to methodological limitations, these estimates are unlikely to be representative of the whole country population. According to Faiz, 1666 snake bite victims attended to the Chittagong Medical College Hospital (CMCH) for treatment between 1993 and 2003. Among those victims, 28.5% were bitten by poisonous snakes and only eight (0.5%) died [1].

In this context, this cross-sectional survey was carried out to determine the annual incidence density of snake bite in rural Bangladesh. In addition, the study also developed an epidemiologic profile of snake bites that includes age and sex specific incidence of snake bites, consequence of snake bite, treatment seeking behaviour of the patients, seasonal trend, and geographical distribution of snake bites in the context of rural Bangladesh. The study was conducted during February to June 2009 in Bangladesh.

## Methods

### Ethics statement

This study was conducted according to the principles expressed in the Declaration of Helsinki. The study was approved by the Human Research Ethics Approval Committee, The University of Newcastle and the Bangladesh Medical Research Council (BMRC). Informed written consent was obtained from head or in his/her absence from any adult member of each selected household.

### Study population

Bangladesh is divided into six administrative divisions. We undertook a multistage cluster sample of households within each administrative division. Firstly, all six administrative divisions were selected. Afterwards, four districts from each selected division, two upazilas from each selected district, two unions from each selected upazila and two blocks from each selected union were randomly selected. At present, the block is the lowest administrative unit in both Urban and rural areas in Bangladesh. The number of households required per block per division was selected based on probability proportionate to size of the total population.

In absence of any sampling frame of households at the block level, we arbitrarily divided each block into four quarters. We then scatteredly identified one-fourth of the required households from each quarter of the block. After selection of the households, interviewers visited all these households and explained the study to the head or in his/her absence any adult member of the household. After obtaining the respondent's written consent, information was collected on socio-demographics, snake bites and their consequences, and treatment seeking behavior following snake bites from the respondent. The respondent answered for every member who spent any part of the past year in the selected household. All information was collected using an interviewer administered pre-tested partially close ended questionnaire through face to face interview. Frequency of snake bite(s) on each member and their length of stay in months in that house during past 12 months were collected from the respondents. Later on, person-time was converted from person-month to person-years to calculate annual incidence density of snake bites in rural Bangladesh.

### Statistical analysis

We estimated the incidence density, and 95% poisson confidence interval of snake bite by using the number of episodes of snake bites as the numerator and person years of stay in the surveyed house as the denominator. Incidence density was adjusted for the design effect of the survey by using “svy” command in STATA. Since there was no non-response in our survey, only design-based weights computed as the inverse of each observational unit's probability of selection at each stage of sampling were used to obtain unbiased estimates of population rates[9]. The chi squared statistic was calculated to compare incidence rates among categories of age, occupation and other variables. We also used this incidence density to estimate the total number of episodes of snake bite per year in rural Bangladesh. We calculated the total number of snake bite victims and related deaths by extrapolating the proportion of snake bite and related death from this study on the total population of rural Bangladesh (BBS 2001).To determine any correlation of snake bite with rain fall and temperature we plotted monthly rate of snake bite against mean monthly rainfall and temperature. All statistical analyses were carried out using Stata version 10.1 [10].

A sample size of 16500 is sufficient to demonstrate an annual incidence of 50 per 100,000 with a 95% confidence interval of ±20 per 100,000. Considering the non-compliance and non-response, an extra ∼10% of the participants were included. Therefore, 3993 households were approached.

## Results

We collected information on 18857 individuals from 3993 households. A sample of 1287, 975, 678, 272, 285 and 496 households were selected from Dhaka, Rajshahi, Chittagong, Barisal, Sylhet and Khulna division, respectively. The total population for Dhaka, Rajshahi, Chittagong, Barisal, Sylhet and Khulna divisions are 39,044,716; 30,201,873; 24,290,384; 8,173,718; 7,939,343; 14,705,223; respectively [11] (BBS 2001). The main characteristics of the participants are given in Table 1.

**Table 1:** Characteristics of the study population* (n = 18,857).

Variable	Frequency (%)
**Sex** *	
Male	9773 (52)
Female	9075 (48)
**Age (years); mean ± SD**	26.75±18.87
**Age category (years)** **	
0–10	4619 (25)
11–20	4032 (21)
21–30	3520 (19)
31–40	2614 (14)
41–50	1925 (10)
51>	2142 (11)
**Religion** **	
Muslim	16854 (89)
Hindu	1728 (9)
Christian	38 (1)
Buddhist	232 (1)
**Occupation** **	
Service	1026 (5)
Farmer	2178 (12)
Student	4546 (24)
Housewife	4766 (25)
Business	1200 (6)
Day laborer	1279 (7)
Others	3857 (21)
**Number of participants per division**	
Barisal	1277 (7)
Chittagong	3602 (19)
Dhaka	5983 (32)
Khulna	2164 (11)
Rajshahi	4272 (23)
Sylhet	1559 (8)
**Years of schooling**	
None	6824 (36)
1–5	6258 (33)
>5	5775 (31)
**Total household monthly income (taka)**	
< = 3000 Tk	6589 (35)
3001–4500 Tk	2888 (15)
4501–7000 Tk	5128 (27)
>7000 Tk	4252 (23)
**Marital status**	
Married	9453 (50)
Unmarried	8825 (47)
Others	579 (3)

*Missing data = 9,

**Missing data = 5.

There were 98 snake bites reported overall, and only one person died of the snake bite. The incidence of snake bite episode was 623.4 (95% CI 513.4–789.2) bites per 100,000 person years. The highest incidence was found for Barisal division (2667.7) and the lowest for Sylhet division (321.6). The between division rates were significantly heterogeneous (Table 2). Eight percent of the snake bite victims are bitten more than once in a year, therefore the total number of snake bite episodes exceeds the number of snake bite victims.

**Table 2:** Distribution of snake bite by division.

Division	Number of snake bites	Annual incidence per 100000 person-years* (95% CI)
Dhaka	22	440 (285–649.9)
Chittagong	9	397.8 (211.8–680.3)
Barisal	22	2667.7 (1787.2–3829.5)
Khulna	20	936.2 (571.9–1445.6)
Sylhet	5	321.6 (104.4–750.2)
Rajshahi	20	472.7 (288–680.3)
Over all	98	623.4 (513.4–789.2)

*: Weighted estimates have been used; P<0.001 from Rao-Scott chi square test (adjusted for sampling design) with 5 degrees of freedom.

Age-specific analysis shows that the percent of victim among the oldest age group (>51) is similar (23%) to that in the young age group of 11–20 years (22%). However, the annual incidence is nearly double (1063 per 100,000) in the old age group because of the small proportion of people at risk under this category (Table 3). Similarly, both of the youngest age group (0–10 years) and the age group of 41–50 account for the 11% of snake bite victims, but the annual incidence in the youngest group (248 per 100,000) is less than half of the other group due to due to the large population at risk in the youngest group.

**Table 3:** Distribution of 98 snake bite by age group.

Age-group	Frequency of victims	Percent of victims	Annual incidence per 100,000*
0–10	11	11	248 (101–394)
11–20	21	22	544 (312–776)
21–30	18	18	531 (286–776)
31–40	15	15	594 (294–814)
41–50	11	11	587 (241–934)
>51	22	23	1063 (621–1506)

*adjusted for sampling design.

Analysis by sex reveals that snake bites are similarly distributed among males (52%) and females (female 48%), the annual incidence density is also similar for males and females(698, 95% CI 538.8–889.6) than females (543.5, 95% CI 399.4–722.7) (not shown in analysis).

Housewives made up the highest category of snake bite victims (30%) with the smallest percentage occurring in day laborers (8%) (Table 4).

**Table 4:** Distribution of 98 snakebite (percent) by occupation, place of bite and activities during the bite.

Occupation	Frequency (%)	Place of bite	Frequency (%)	Activities during the bite	Frequency (%)
Housewife	29 (30)	Water	26 (27)	Lying/sleeping	15 (15)
Student	20 (21)	Field	24 (24)	Walking	28 (29)
Farmer	19 (19)	Road side	22 (23)	Working in field	18 (18)
Businessman	11 (11)	Inside home	12 (12)	Fishing	14 (14)
Day laborers	8 (8)	Home premises	11 (11)	others	23 (24)
Others	11 (11)	Others	3 (3)		

A relatively high proportion of snake bite episodes happened during night times (36%), whereas morning and afternoon account for same proportion (32%) close to a third in each category. Housewives receive more bites (40%) at night where as farmers receive more bites (71%) during day time. Bites that occur at night are more frequent at home or on the road side (59%) (not shown in analysis).This is perhaps related to krait being an important snake in Bangladesh, and would explain the increased proportion of housewife victims as well.

Most of the snake bites occurs in water (27%) followed by the field (24%). Home premises and inside the home account for similar proportion of bites (11% and 12% respectively) (Table 4).

As expected, the majority of snake bites occurred on the feet (71%), followed by the hand (27%) and other parts of the body (2%) (not shown in analysis).

Although highest proportion of snake bite occurs in water (27%), fishing accounts for the lowest proportion (14%) of all activities (Table 4). This may be because during monsoon season, many people travel by boat as the roads are often submerged under water.

**Figure 1:**
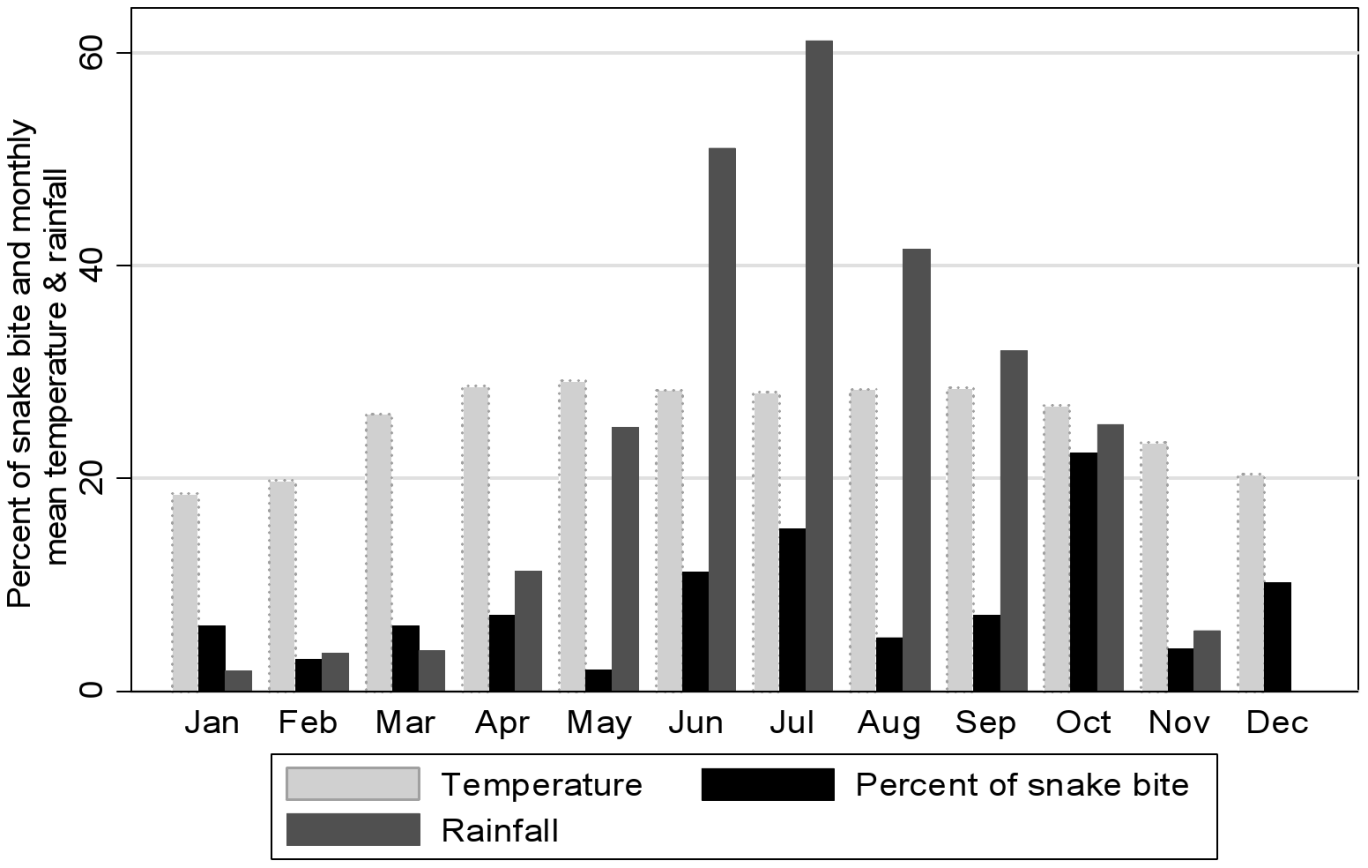
Distribution of snake bites, temperature and rain by month.

The distribution of monthly snake bites, rainfall and temperature data are presented in Figure 1. The month of October (22%) and July (15%) account for the highest proportion of bite episodes. There is also an increase in snake bites in the month of December as harvesting activities increase during this month. Although scatter plot of monthly snake bite rate and mean rainfall and temperature did not show any evidence of linear relationship.

After recognizing a snake bite episode the victims received different combination of management strategies. The distribution of post snake bite management options are shown in Table 5. Since most of the victims received more than one form of management, the total percentage is great than 100%. The most prevalent management is recitation of mantras by the snake charmers (60%) or ‘ozhas’. Potentially harmful approaches such as making multiple incisions around the bite site, incorrect application techniques in tourniquets (e.g. wrong pressure), sucking blood orally from the multiple cuts are practiced in an alarmingly high proportion of cases. In only 31% of snake bites the bitten parts of the body were kept immobile, which is likely to be clinically beneficial to limit systemic spread of venom.

**Table 5:** Distribution of treatments received by the 98 snake bite victims.

Type of treatment	Frequency of victims	Percent of victims
1 tourniquet was given	37	39
>1 tourniquets were given	26	27
Kept immobile	30	31
Sucked blood orally	10	11
Multiple cuts around the bite site	41	43
Utters mantras	57	60
Forced vomiting	17	18

Snake charmers were the first contact following a snake bit for 86% of the victims, with 6% contacting traditional village doctors, only 3% contacting a registered physician or a nearby hospital and the remaining 5% contacting other sources such as directly going to a drug store (not shown in analysis). This information is consistent with Table 5 that almost all the treatments were offered by the traditional healers, i.e., ozhas or snake charmers.

## Discussion

We have assessed the risk of snake bite in rural Bangladesh. The findings suggest that an annual incidence density of snake bite is 623.4 per 100,000 person-years, which may be as high as 789.2 per 100,000 person-years. According to the study findings an estimated 710,159 episodes of snake bites occur annually in rural Bangladesh. An estimated 589,919 individuals are bitten by snakes and 6041 die from snake bites every year in rural Bangladesh. This incidence is much higher than the previously estimated incidence. The highest incidence was observed in southwestern coastal Barisal division followed by Khulna division. The highest incidence was also found in Barisal division previously [7]. This high rate of Barisal division could be attributed to its geographical location and natural environmental condition.

In this study, the majority of the snake bite victims are of a younger age and this reflects that an active population is at higher risk of snake bites. Similar observations were reported from Nepal, Malaysia and a previous study in Bangladesh [7], [8], [12], [13], [14], [15]. This information has important public health implications that despite of the comparatively low incidence the youngest age group should be given priority in directing any intervention for snake bite. Our study found a similar ratio of male and female snake bite victims. Male preponderance was observed from a few studies, largely due to bites in paddy fields although male to female ratio varied in these studies [7], [8], [12], [16], [17], [18]. Our study finding on the male female ratio differs from other studies. This difference may partly be explained as this is a community based study compared to most of the reported hospital based studies and therefore more likely to be representative of the total population exposed. Males may have higher hospitalization rates than females in developing countries. Moreover, women receive more bites at home and during night time. This may be due to the presence of krait at the home premises. Krait is usually nocturnal. It lives close to the human dwellings and hides in holes, woods or homestead gardens. At night, especially at the height of the monsoon season, kraits enter into human dwellings, presumably in hunt of their natural prey that includes small snakes, amphibians, rodents, and geckos, which are abundant in rural homes [19], [20], [21]. In many areas of Bangladesh, women are also involved in agricultural activities at the field with the males.

In our study, we found that the highest number of snake bites occurs during the monsoon season from June to October. This is probably because most of the agricultural activities take place during this season. Furthermore, there is increased snake activity during this period in the hot weather and monsoon's rain. These changed conditions are likely to force snakes to come out of their shelters and seek refuge in comparatively high and dry places. This may be partially responsible for increased risk of snake bite during the monsoon season. Similar findings were reported from other studies.[7], [8], [12], [22], [23]. An increase of snake bite in December was also observed. This is likely due to increased agricultural activities in this month. Our data did not suggest any relationship between monthly snake bites, temperature and rainfall. Even if there is any relationship between snake bites, temperature, and rainfall, often it is difficult to determine exactly what the relationship is, particularly if there are two things that are driving the effect, e.g. rainfall and temperature or rainfall and harvesting.

Biting occurs mostly when individuals are at work, engaging in activities such as cultivation, fishing, plantation, wood collection, or tending crops or gardens. However, bites are fairly common when the victims are walking or sleeping. In this study, 51% of victims received their snake bite while working either in the agricultural field or in water. Twenty three percent had snake bites while staying at home. Most of the houses in Bangladesh are not brick and the snakes sometimes live in the holes of the muddy floors. Moreover, most of the houses in rural areas of the country have homestead bush, which offers normal habitats for snakes. As a result, events of snake bites are also common when people are at home. To go to the toilet and for other domestic purposes, people often come out of their houses and become victims. Village people store grains, including paddy, in their bedroom, which also provides shelter to the snakes, therefore increasing the risk of snake bite. Similar observations were also reported from a previous study in Bangladesh [7].

The majority of the victims (71%) received snake bites in lower extremities. This may be because most of the time the snakes were trodden upon by the victims. Bites at the agricultural fields are also more likely to occur on the lower limbs. Cobras are common sources of daytime bites in Bangladesh. Similar findings were reported from Nepal, Bangladesh, Malaysia and Hong Kong [7], [8], [12], [13], [14], [24], [25]. Although Plowman and co-workers reported that two-thirds of the snake bite cases occurred in the upper limb [26], in our study, 27% victims received snakebites at their upper limbs. Many of the bites just occurred while lying on the ground in bed. Bites in the upper limbs or head and neck region may occur during sleep on ground as kraits often enter human dwelling at night in search of food. Similar observations were made in Nepal earlier [12], [27]. Bites in the head and neck region may also occur by the green pit viper which was also found in earlier studies in Chittagong area [1].

Seventy five percent of the victims received any form of management within two hours of the snake bite. Eighty six percent of the victims go to a snake charmer first to seek treatment, only three percent go to a medical doctor or hospital directly after the bite. Later on, 10% victims visit a medical doctor or hospital. He majority of the rural people do not want to go to a doctor following snake bites. The reasons for this require thorough evaluation but may include lack of awareness of the efficacy of medical treatment with antivenins, lack of availability of snake antivenins in the public hospital, lack of transport facilities and inability to afford transportation. Similar observations were made in the past studies conducted in Nepal [12], [18], [27].

Intravenous snake antivenin is the most effective treatment for envenoming by snakes [28]. Our study observed that snake charmers practice many unhygienic measures such as multiple incisions, tight tourniquet around the bite mark, sucking of blood from the bite wound to manage the snake bite. Therefore, these snake charmers should be trained on as a priority, so that they can stop their risky practices, perhaps be trained to apply tourniquets correctly and immediately refer the patients to the nearest health facilities. Snake antivenin should be made available in the public hospitals free of cost, particularly in the remote rural areas.

To the best of our knowledge, this is the largest community based study so far that has been conducted to the determine incidence of snake bites in any country in the world. The main strength of this study is its epidemiological approach which was followed rigorously at each stage of the study. Limitations of this cross-sectional study involve the methods that were used for sampling at the block level. Because the survey involved remote rural areas and because no list of households exists in those areas, a perfectly random sample could not be obtained. However, there is no reason to believe that the adopted sampling strategy would have resulted in a non-representative study population. We collected information from the respondents on snakebite which occurred during the previous twelve months. Although recollecting information from the past, recall error is unlikely to occur because the victim or the household members are less likely to forget an important event such as snakebite. Households that were fully vacant during interview team's visit were excluded from the survey. We did not record the actual number of vacant houses. However, only a very few households were totally vacant during the household survey. In this survey, we observed only one death out of 98 snakebites; this may reflect the nature of snake bites in Bangladesh, the majority of which are non-venomous. This study reported that only one person died of the snake bite. Based on this single death, we estimated the number of snakebite related deaths i.e., 6041 in rural Bangladesh. Although this single death may not be statistically stable to estimate total snake bite related deaths, the estimated number of deaths seem reasonable. The estimated number of deaths is also likely to be representative because of this large population based representative survey. We only collected information on annual incidence of snake bites in rural Bangladesh in this study. Future studies could investigate snakebites which occurred throughout the life of study participants, although recall bias is likely to increase with increasing time since the event.

The study findings would be useful for planning and formulating strategies and specific interventions to combat snake bite related health problems in Bangladesh. Poor access to health services increases the risk of morbidity and mortality attributable to snake bites. Scarcity of supply of snake antivenin is a major factor which needs to be addressed by local production. Snake bite related deaths are preventable.

## Supporting Information

Checklist S1 STROBE checklist.
(0.08 MB DOC)
